# Is day-case surgical procedure safe for MICRA leadless pacemaker implantation?

**DOI:** 10.1007/s10840-024-01907-7

**Published:** 2024-08-19

**Authors:** Lin-Thiri Toon, Mohammed ElRefai, Mohamed Abouelasaad, Roopa Patil, John Paisey, Arthur Yue, Paul Roberts

**Affiliations:** 1 Cardiac Rhythm Management Research Department, University Hospital Southampton, Southampton, SO16 6YD UK; 2 University of Southampton, Southampton, UK

**Keywords:** MICRA, Pacemaker, Patient management, Day-case surgery

## Abstract

**Background:**

MICRA implantation is not commonly done as a day-case procedure. Elective leadless pacemakers are implanted routinely in our centre.

**Objective:**

To assess whether the day-case MICRA procedure is safe.

**Methods:**

We retrospectively collected data from all patients undergoing elective MICRA implantation at our centre between May 2014 and Nov 2022 (*n* = 81). Two patient groups were stratified: those planned to be discharged on the same day (SD, *n* = 52) and those planned to be observed overnight after the procedure (ON, *n* = 29). Patient demographics, size of the sheath used, type of MICRA device, and rate of complications were recorded. In patients with successful implants (*n* = 80), device function at discharge and first routine follow-up were evaluated.

**Results:**

There were 58% males in the SD group and 45% in the ON group. Median age was 49 years in the SD and 67 years in the ON. Among patients who were planned as a day case, 8 patients had to stay in the hospital but for < 48 h: 2 due to minor groin bleeding, 1 due to patient’s request despite fit to discharge, 4 due to the procedure carried out later in the day, and 1 for observation due to procedural complexity. MICRA implantation was successful in 80 patients. The rate of the major complications was 2% in the SD group and 7% in the ON group (*p* = 0.223), and none of the co-morbidities assessed showed an association with any complications. Device parameters at the follow-up were available in 76 patients. The rate of patients with low and stable PCT at follow-up was also 98% in the SD group and 96% in the ON group.

**Conclusions:**

Day case MICRA procedure can be performed safely in an appropriately selected patient population.

**Graphical abstract:**

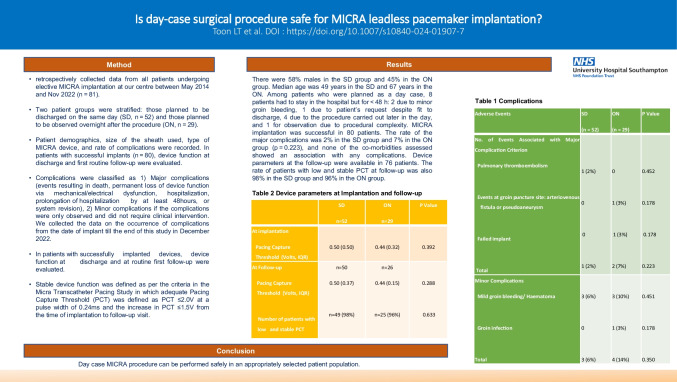

## Background

Implantation of cardiac implantable electronic devices (CIEDs) is one of the most common cardiac procedures. Worldwide over 1.25 million pacemakers are implanted each year [1]. Day-case CIED procedures are considered cost-effective and safe and hence are routinely done in many healthcare institutions [2, 3]. A leadless pacemaker is a relatively new cardiac device, and it is designed to avoid complications related to subcutaneous pockets and transvenous leads. Leadless pacemakers are not commonly implanted as a day-case procedure in current practice. However, elective day-case leadless pacemakers are implanted routinely at our centre. In this study, we present data on the largest population in the medical literature and evaluate the safety of our elective leadless pacemaker implantation practice in this study.

## Methods

### Patient selection

We retrospectively collected data from all patients undergoing MICRA leadless pacemaker implantation at University Hospital Southampton between May 2014 and November 2022 (*n* = 139).

### Data collection

We collected patient demographic data (age, sex, co-morbidities) and parameters related to the procedure including indication for pacing, size of the introducer sheath, and type of MICRA pacemaker. All data were obtained from the hospital’s medical electronic records. We have included all the patients who underwent MICRA implantation at our tertiary hospital since 2014.

We also included information regarding the rate of procedure-related complications. Complications were classified as (1) major complications (events resulting in death, permanent loss of device function via mechanical/electrical dysfunction, hospitalisation or prolongation of hospitalisation by at least 48 h, or system revision) and (2) minor complications if the complications were only observed and did not require clinical intervention. We collected the data on the occurrence of complications from the date of implant till the end of this study in December 2022.

In patients with successfully implanted devices, device function at discharge and routine first follow-up was evaluated. Stable device function was defined as per the criteria in the Micra Transcatheter Pacing Study in which adequate pacing capture threshold (PCT) was defined as PCT ≤ 2.0 V at a pulse width of 0.24 ms and the increase in PCT ≤ 1.5 V from the time of implantation to follow-up visit [4].

### Data analysis

At our centre, MICRA implantation procedures were started in early 2014 and elective out-patient MICRA implantations were started in early 2015. In this study, the data from elective MICRA patients were collected. Two patient groups were identified: those planned to be discharged on the same day (SD) and those planned to be observed overnight after the procedure (ON). The rate of major and minor complications was compared between the two groups.

Between 2014 and 2022, 139 patients underwent the procedure. Among them, 81 patients were elective outpatients (SD, *n* = 52, and ON, *n* = 29). Fifty patients in the SD group and 26 patients in the ON group had follow-up device parameters available for analysis.

### Statistical methodology

IBM SPSS Statistics Version 29.0.1.0 was used for statistical analysis. Data distribution was assessed using histograms, Q-Q plots, box plots, and normality tests. Categorial data were represented as *n* (%) and non-parametric continuous data as median (IQR). The data were tested using the Mann–Whitney *U* test and Pearson chi-square test.

## Results

### Baseline and procedure-related characteristics

We have enrolled 81 patients who underwent elective MICRA leadless pacemaker implantation during the study period (Table 1). Among these patients, 52 patients were planned to be discharged on the same day after the procedure (SD, *n* = 52), and 29 patients were planned to be observed overnight (ON, *n* = 29). Patients in the SD group were younger compared to the patients in the ON group (49 vs 67, *p* = 0.082). In the SD group, 58% are male while 45% of the ON group are male (*p* = 0.266). The distribution of co-morbidities is comparable between the two groups.

**Table 1:** Baseline characteristics and indications for pacing

	SD *n* = 52	ON *n* = 29	*p* value
Age (years)^1^ Sex (male, *n*) Co-morbidities (*n*) Hypertension Diabetes Heart failure Coronary artery disease Stroke/TIA Congenital heart disease Peripheral arterial disease COPD CKD ESRD Dialysis History of infective endocarditis History of bacteraemia	49 (32) 30 (58%) 10 (19%) 5 (10%) 4 (8%) 2 (4%) 6 (12%) 3 (6%) 1 (2%) 3 (6%) 6 (12%) 0 0 2 (4%) 4 (8%)	67 (39) 13 (45%) 7 (24%) 3 (10%) 0 4 (14%) 0 2 (7%) 1 (3%) 1 (3%) 1 (3%) 1 (3%) 1 (3%) 0 1 (3%)	0.082^2^ 0.266^3^ 0.603^3^ 0.916^3^ 0.126^3^ 0.101^3^ 0.057^3^ 0.840^3^ 0.672^3^ 0.644^3^ 0.214^3^ 0.178^3^ 0.178^3^ 0.285^3^ 0.447^3^
Indications for pacing AF with pause AVN ablation High-grade AV block Junctional bradycardia Myotonic dystrophy Sinus node dysfunction Symptomatic pauses Symptomatic slow AF Tachy-Brady syndrome Trifascicular block + syncope Ventricular standstill	4 (8%) 5 (10%) 10 (19%) 1 (2%) 2 (4%) 2 (4%) 22 (42%) 0 2 (4%) 1 (2%) 3 (6%)	4 (14%) 2 (7%) 10 (34%) 0 1 (3%) 1 (3%) 9 (31%) 2 (7%) 0 0 0	0.378^3^ 0.676^3^ 0.127^3^ 0.452^3^ 0.928^3^ 0.928^3^ 0.317^3^ 0.055^3^ 0.285^3^ 0.452^3^ 0.187^3^
Type of Micra Micra VR Micra AV	*n* = 52 38 (73%) 14 (27%)	*n* = 28 25 (89%) 3 (11%)	0.098^3^ 0.173^3^ 0.079^3^

^1^Median (IQR), ^2^Mann-Whitney *U* test, ^3^Pearson chi-square test

In terms of indications for pacing, SD patients received leadless pacemakers mainly due to symptomatic pauses while high-grade atrioventricular (AV) block is the commonest indication among the ON group. For delivery of the MICRA system, an ultrasound-guided transvenous venous access and 23 French inner diameter/27 French outer diameter sheath were used in all patients.

At our centre, Z-suture and manual compression for 15 min are used as a standard for haemostasis after the procedure. Patients are kept in the supine position for 2 h followed by another 2 h in a 45° sit-up position before suture removal and gentle mobilisation.

### Procedural outcomes

In 80 out of 81 patients, the leadless pacemaker was successfully implanted. The procedure had to be abandoned for only one patient in the ON group. That patient was a patient with complex congenital heart disease and a very dynamic right ventricle. Multiple attempts were required to achieve satisfactory stability. However, the device had dislodged soon after the tether was cut. Hence, the implant success rate was 100% in the SD group and 97% in the ON group. Overall, the MICRA VR device was implanted in 63 patients and the MICRA AV device was implanted in 17 patients.

**Fig. 1:**
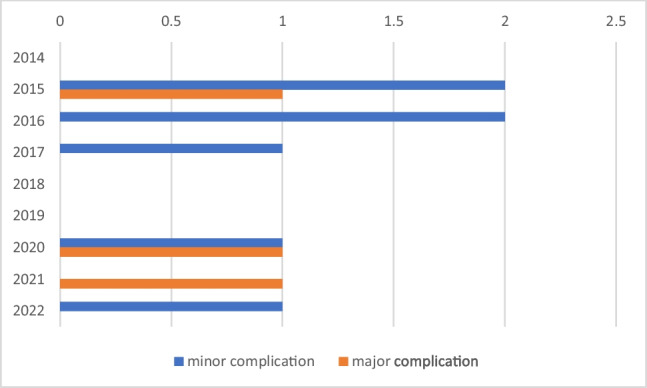
Distribution of the incidence of complications through time

Among SD group patients, 8 patients had to stay in the hospital but for < 48 h: 2 due to minor groin bleeding, 1 due to patient’s request despite fit to discharge, 4 due to procedure carried out later in the day, and 1 for observation due to procedural complexity. Forty-four patients in this group were successfully discharged on the same day. All patients in both study groups had sufficient follow-up data to evaluate the rate of complications. The rate of procedure-related complications was similar between the two groups (Tables 2, 3, 4). None of the co-morbidities had a significant association with any complications. The incidence of complications over time is shown in Fig. 1.

**Table 2:** Complications

Adverse events	SD (*n* = 52)	ON (*n* = 29)	*p* value^1^
No. of events associated with major complication criterion			
Pulmonary thromboembolism Events at groin puncture site: arteriovenous fistula or pseudoaneurysm Failed implant Total	1 (2%) 0 0 1 (2%)	0 1 (3%) 1 (3%) 2 (7%)	0.452 0.178 0.178 0.223
Minor complications			
Mild groin bleeding/haematoma Groin infection Total	3 (6%) 0 3 (6%)	3 (10%) 1 (3%) 4 (14%)	0.451 0.178 0.350

^1^Pearson chi-square test

**Table 3:** Co-morbidities vs minor complications

Minor complications			
Co-morbidities	Minor groin bleeding/haematoma (*n*)	Groin infection (*n*)	*p* value^1^
Hypertension Diabetes Stroke/TIA Peripheral arterial disease COPD	1 1 2 1 1	0 0 0 0 0	0.659 0.659 0.495 0.659 0.659

^1^Pearson chi-square test

**Table 4:** Co-morbidities vs major complications

Major complications				
Co-morbidities	AV fistula or pseudoaneurysm (*n*)	Pulmonary thromboembolism (*n*)	Failed implant (*n*)	*p* value^1^
Hypertension Diabetes Congenital heart disease CKD	1 1 0 0	0 0 0 1	0 1 1 1	0.223 0.223 0.223 0.223

^1^Pearson chi-square test

### Adverse events associated with major complication criterion

One patient in the SD group started to suffer from pulmonary symptoms around 2 weeks after the procedure and was diagnosed with pulmonary embolism.

MICRA device was dislodged soon after deployment in one patient among the ON group. The device was successfully retrieved using snarls, and the procedure was abandoned. Another patient in the ON group developed an arteriovenous fistula and required surgical intervention.

### Minor complications

Mild groin bleeding or haematoma had occurred in three patients in each group. One patient in the ON group was found to have a mild groin infection after the procedure.

### Device function at implantation and follow-up

Among the elective patients who successfully underwent the procedure, device parameters at routine first follow-up were available for analysis in 50 patients of the SD group and 26 patients of the ON group (Table 5).

**Table 5:** Device parameters at Implantation and follow-up

	SD *n* = 52	ON *n* = 29	*p* value
At implantation Pacing capture threshold (volts, IQR)^1^	0.50 (0.50)	0.44 (0.32)	0.392^2^
At follow-up Pacing capture threshold (volts, IQR) Number of patients with low and stable PCT	*n* = 50 0.50 (0.37) *n* = 49 (98%)	*n* = 26 0.44 (0.15) *n* = 25 (96%)	0.288^2^ 0.633^3^

^1^Median (IQR), ^2^Mann-Whitney *U* test, ^3^Pearson chi-square test

The rate of patients at low and stable pacing capture threshold at follow-up was 98% in the SD group and 96% in the ON group.

## Discussion

In the last few decades, a combination of new surgical techniques, advances in anaesthesia, and pain control have led to a switch to day-case surgery. Day surgery is a safe and cost-effective approach to surgical health care. In 2013–2014, the average cost for day-case surgical procedures in the UK was £698 ($842.58) and the average elective inpatient care cost was £3375.50 ($4074.66) [5]. The increasing proportion of day-case procedures has helped reduce overall costs. Many other advantages of day surgery are shortening hospital stays, increasing hospital bed availability, and decreasing waiting lists. Day surgery also carries fewer risks of hospital-related infections.

European Heart Rhythm Association (EHRA) conducted a survey in 68 EHRA EP research network centres in 2014 [6]. This survey evaluated all cardiac device implantations including implantable cardioverter defibrillators (ICD) and cardiac resynchronisation therapy (CRT). 18.30% of the centres performed planned cases as day-case admissions. Forty-seven percent of the centres do a single overnight stay for device procedures, while 8.13% have two-night admissions and 10% more than two nights. The reasons for this diversity may include financial reimbursement of day surgical cases, regulations and incentives in different countries, and individual practices of operators and anaesthetists.

MICRA leadless pacemaker is a single-chamber ventricular pacemaker, and it has a volume of 0.8 cm [3], a length of 25.9 mm, an outer diameter of 6.7 mm, and a weight of 2.0 g [7]. The device sits in a cup at the distal end of a steerable transfemoral catheter delivery system and is delivered through the femoral vein using a 23 French internal diameter/27 French outer diameter introducer. The steerable catheter with preloaded MICRA is advanced into the right ventricle, and the device is deployed by fixation into the myocardium using the associated protraction of the nitinol tines. After confirmation of device fixation and electrical measurements, a tether is cut, and the delivery system is removed. One of the concerns regarding the safety of the day-case MICRA procedure is related to the usage of 27 French outer diameter introducers.

To the best of our knowledge, this is the largest patient population undergoing MICRA leadless pacemaker implantation as a day-case procedure. Kiani et al. [8] evaluated the safety and feasibility of the day-case MICRA procedure at their institution and reported that same-day discharge after the MICRA procedure is safe in appropriately selected individuals. In the Kiani et al. study, only 25 patients underwent MICRA implantation as a day-case procedure. In our study, 52 out of 139 patients were planned day-case patients, and 44 patients were successfully discharged on the same day. The aim of this study was to evaluate the safety of our elective leadless pacemaker implantation practice. The results as detailed above show a high implant success rate (99%) and a stable device function at follow-up at 98% and 96% in each group (*p* = 0.633). The rate of major procedure-related complications in the two groups was 2% vs 7%, *p* = 0.223.

Patients in the same-day discharge group in our study were notably younger than patients in the overnight observed group. It means that the same-day discharge group possibly included young, active, and less unwell patients. This data suggested that same-day discharge after MICRA implantation is safe in carefully selected patient cohorts.

Compared to most previous MICRA studies [4, 7–10], our MICRA population is significantly younger. This is largely related to the restrictions imposed by the National Institute of Health and Care Excellence (NICE, UK) in 2018. NICE (UK) recommends performing MICRA leadless pacemaker implantation only as a part of research or only when the conventional transvenous pacemaker is contraindicated and a multidisciplinary team (MDT) determines that MICRA device should be used [11]. On this basis, many young patients who require pacing may be considered by an MDT to have a contraindication to transvenous leads owing to the complications associated with leads, in the vasculature, over many years.

**Fig. 2:**
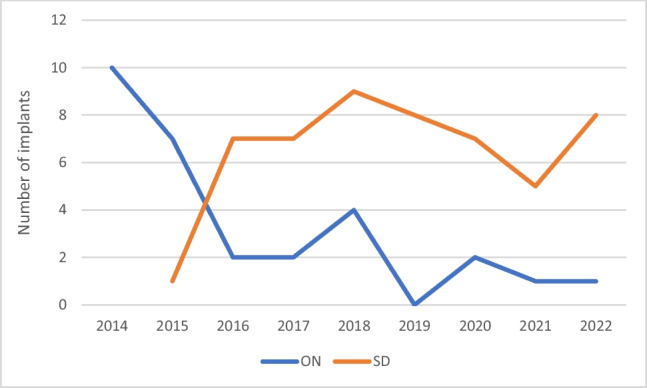
Number of MICRA implants at our centre since 2014 (ON = observed overnight group, SD = same-day discharge)

At our centre, MICRA implantation was started in early 2014 (Figs. 1 and 2). Patients undergoing early MICRA implantation at our centre were inpatients or were scheduled for admission after the procedure. Day-case MICRA implants were started only in 2015 when the clinical team had achieved enough exposure and experience and the number of patients who stayed overnight in the hospital after the procedure had come down subsequently. Day-case MICRA implantation has become the standard for our elective out-patients unless the patient has complex anatomy or other social needs to stay in the hospital after the procedure. Although it can make an impact on the rate of complications and device function, the rate of complications and stable device function at follow-up are similar in both patient groups. The implant success rate in the same-day discharge group was 100%, and 98% of these patients remained on a stable pacing capture threshold at the first routine follow-up in our study. These findings were better than previously reported data in previous MICRA studies [4, 9]. The rate of major complication was 2% in the same-day discharge group, and this finding was consistent with the MICRA Investigational Device Exemption (IDE) study [4]. Apart from mild groin bleeding, there was no major groin puncture site complication occurring in the same-day discharge group.

## Study limitations

There are several limitations to our study. Firstly, a randomised controlled trial is required to prove the non-inferiority or superiority of day-case procedure to hospitalised procedure, but our patients were not randomised, and it is possible that patients with the lowest risk were selected for the day-case procedure. Secondly, the data presented is based on a small number of patients, and thus, the chance of detecting rare complications was limited and the number of complications was not high enough to make a statistical significance.

## Conclusion

Hospital admission for cardiac device procedures can put a burden on medical resources. Our study suggests that day-case surgery for MICRA leadless pacemaker implantation can be performed safely in a carefully selected population under the care of an experienced clinical team.

## Data Availability

The data that support the findings of this study are available from the authors upon reasonable request and with permission from the University Hospital Southampton NHS Foundation Trust.
